# Impact of New vs. Old International Children’s Continence Society Standardization on the Classification of Treatment Naïve Enuresis Children at Screening: The Value of Voiding Diaries and Questionnaires

**DOI:** 10.3389/fped.2022.862248

**Published:** 2022-03-28

**Authors:** Sevasti Karamaria, Nadejda Ranguelov, Pernille Hansen, Veerle De Boe, Pieter Verleyen, Nathalie Segers, Johan Vande Walle, Lien Dossche, An Bael

**Affiliations:** ^1^Department of Internal Medicine and Pediatrics, Ghent University, ERKNET, Ghent, Belgium; ^2^Department of Pediatrics, Cliniques Universitaires St-Luc, Université catholique de Louvain, Brussels, Belgium; ^3^Department of Pediatrics, CHU Tivoli, La Louvière, Belgium; ^4^Department of Urology, Brussels University Hospital, Brussels, Belgium; ^5^Department of Urology, AZ Groeninge, Kortrijk, Belgium; ^6^Department of Pediatrics, Pediatric Nephrology, Hospital Network Antwerp (ZNA) Koningin Paola Kinderziekenhuis, Antwerp, Belgium; ^7^Department of Pediatric Nephrology, Ghent University Hospital, Ghent, Belgium; ^8^Faculty of Medicine, University of Antwerp, Antwerp, Belgium

**Keywords:** children, nocturnal enuresis, clinical management tool, questionnaire, diary, screening, MNE, NMNE

## Abstract

Expert consensus papers recommend differentiating enuresis using questionnaires and voiding diaries into non- (NMNE) and monosymptomatic enuresis (MNE) is crucial at intake to decide the most appropriate workout and treatment. This national, Belgian, prospective study investigates the correlation, consistency, and added value of the two methods, the new against the old International Children’s Continence Society (ICCS) definitions, and documents the prevalence of the two enuresis subtypes in our population. Ninety treatment-naïve enuretic children were evaluated with the questionnaire, and the voiding diary and the two clinical management tools were compared. Almost 30% of the children had a different diagnosis with each method, and we observed inconsistencies between them in registering Lower Tract Symptoms (κ = –0.057–0.432 depending on the symptom). Both methods had a high correlation in identifying MNE (*rs* = 0.612, *p* = 0.001) but not for NMNE (*rs* = 0.127, *p* = 0.248). According to the latest ICCS definitions, the incidence of MNE was significantly lower (7 vs. 48%) with the old standardization.

**Conclusion:**

The voiding diary and the questionnaire, as recommended by the ICCS at the screening of treatment-naïve enuretic patients, are considerably inconsistent and have significantly different sensitivities in identifying LUTS and thus differentiating MNE from NMNE. However, the high incidence of LUTS and very low prevalence of MNE suggest that differentiating MNE from NMNE to the maximum might not always correlate with different therapy responses.

## Introduction

Where pathogenesis of nocturnal enuresis (NE = bedwetting) was until the last decades considered as a benign retarded maturation defect of bladder control, at present, the majority of authors agree that bedwetting coincides with a mismatch between nocturnal diuresis volume and functional bladder volume overnight, paired with an inability to arouse from sleep (1–3) and a genetic predisposition. Since the 80s, first-line treatments included cognitive training with or without enuresis alarm (4, 5), which was considered a conditioning method. The introduction of desmopressin, a synthetic vasopressin analog with an antidiuretic action, had promising results with a 60% success rate (6), but later studies in unselected populations documented less than one-third success rates. Moreover, desmopressin was mainly effective in enuretics with frequent nocturnal polyuria (7). Children responded better to the alarm and to desmopressin if they did not have daytime incontinence (8), and up to 30% did not become completely dry with these methods (9, 10). The beneficial effect of bladder-directed anticholinergic drugs on daytime symptoms in children (11) added evidence for the heterogeneity in pathogenesis. Norgaard et al. (6) concluded that there is a need for a new diagnostic and therapeutic strategy in enuresis patients to identify the majority of patients without daytime symptoms (monosymptomatic enuresis, MNE) who would likely respond to desmopressin and/or alarm and would easily be treated in primary care, avoiding unnecessary burden and examinations. In contrast, in patients with daytime incontinence (non-monosymptomatic enuresis, NMNE), evidence of successful therapy regimens was low, and expert treatment in the secondary line was defendable (12). Daytime incontinence in clinical history was initially the only symptom differentiating NMNE from MNE, but other Lower Urinary Track symptoms (LUTS), namely urgency, voiding difficulties, and abnormal daytime voiding frequency, documented in clinical history and/or diary, were included progressively in the more recent ICCS standardization papers (2, 3, 13, 14). However, the correlation of LUTS other than daytime incontinence with the severity of therapy resistance to the first-line therapy remains unclear.

ICCS recommends differentiating MNE from NMNE at the screening by taking a thorough history, performing a clinical examination, and using voiding diaries (a 2-day daytime diary of fluid intake, voiding volumes, and possible incontinence incidents, as well as a 1-week nighttime diary of the nocturnal urine production) (1, 15). The criteria do not include functional bladder volume and diary registration during normalized fluid intake. Various questionnaires have been developed to help with the medical history procedure. Vande Walle et al. published a practical consensus paper as a supplement to ICCS recommendations (15), with guidelines for screening treatment-naïve enuresis patients. This paper included the use of the Clinical Management Tool (CMT), a checklist with all information needed to be obtained, as mentioned in the standardization documents of the ICCS.

The voiding diary and the CMT can lead to a more objective characterization of enuresis by identifying LUTS. Moreover, the voiding diaries provide valuable information regarding the voiding volumes and help detect nocturnal polyuria. However, the two modalities do not always agree on the diagnosis. There is no consensus if MNE should be excluded if LUTS is present in both questionnaire and diary or only one of the two. This paper aims to study their consistency and added value in identifying LUTS and subtyping enuresis, something that has not been investigated thoroughly, and not their contribution regarding the choice of the treatment based on voided volumes and nocturnal diuresis. Furthermore, we wanted to explore the prevalence of MNE in our population and its characteristics based on the old definition of NMNE (presence of daytime incontinence) and the new one (presence of LUTS with or without daytime incontinence), as older studies, especially in primary care, are often not aligned with the latter.

## Materials and Methods

We performed a national, multicenter, prospective, observational study in children aged 5–16 years, who were primarily consulting for enuresis and treatment naïve: they had never consulted a clinician, nor had ever received any treatment for enuresis. They were seen in the outpatient clinic of seven Belgian Hospitals (three University and four non-University enuresis expertise centers) by a pediatrician, a pediatric nephrologist, or a pediatric urologist. Power calculation showed that we needed 100 naïve enuresis children for our study. Informed consent of both parents or their legal representatives was obtained before inclusion and assent from children older than 12 years old. Ethics Committees of all seven participating hospitals approved the study (Belgian registry number B403201630372).

During the first study visit, a thorough medical history was taken and basic assessments were performed, including physical examination, blood pressure measurement, and a urine dipstick. Children with abnormalities in a urine dipstick, signs of spinal dysraphism, palpable fecaloma, continuous incontinence for urine and/or stool, or hypertension [blood pressure > 90th percentile for age and height according to tables from www.uptodate.com (16)] were excluded. Children with milder forms of constipation, according to the Rome III criteria, were able to enter the study. Furthermore, a questionnaire (Table 1) based on the Clinical management Tool translated into Dutch and French was obtained (15). At the end of the visit, a voiding diary was explained and given to the parents. All voiding and drinking volumes in 2 days were registered. The voiding diary was assessed at the second study visit; if the micturition frequency was ≥ 8 or ≤ 3 per day and/or LUTS as defined by the ICCS (2) was present, a patient was diagnosed with NMNE. We also correlated this *post-hoc* classification with the clinical diagnosis mentioned in the patient file. In addition, we used the old and new definitions of enuresis and incontinence [before and after the first standardization document of ICCS, respectively (17)] to divide our study population according to the diagnosis at visits 1 (based on the CMT) and 2 (based on the voiding diary): (a) MNE/MNE, (b) NMNE/NMNE, (c) MNE/NMNE. Subsequently, we compared the groups for all variables registered in CMT and the voiding diary. We also registered the demographic data of our population (age and sex).

**TABLE 1:** Questionnaire based on CMT (15).

Nocturnal enuresis	Yes	No
Does the child wet the bed?		
Age ≥ 5 years old?		
**Alarm signs and symptoms**		
**Bladder symptoms**	**Yes**	**No**
Leakages of urine during the day (drops of urine in the underpants, leaks in underpants, very wet underpants)?		
History of daytime incontinence after the age of 5 years old?		
Voiding frequency > 8 x/day?		
Voiding frequency < 3 x/day?		
Sudden and urgent need to urinate?		
Push to urinate?		
Several voids, one after another?		
History of uropathy?		
**Infections**	**Yes**	**No**
History of urinary tract infections?		
**Stool**	**Yes**	**No**
Constipation?		
Traces of feces in the underpants?		
**Drinking behavior**	**Yes**	**No**
Drinks a lot in the evening?		
**Psychology**	**Yes**	**No**
Known behavioral or psychological problems (e.g., ADHD)?		

Statistical analysis was performed using SPSS v.26 (IBM Corp., Released 2019. IBM SPSS Statistics for Windows, Version 26.0; IBM Corp., Armonk, NY, United States); we used the Shapiro–Wilk test to check the normality of the variables and (non)parametric tests (Mann–Whitney *U*, Kruskal–Wallis, Wilcoxon rank/McNemar, and Spearman/Pearson correlation rank tests) as applicable.

## Results

A total of 109 patients were included in the study. Nineteen patients were drop-outs. The data of 90 children were successfully processed. The mean age of the children was 7.7 years (±2.0); 62 were boys (69%), and 27 were girls. Out of 90 children, 26 (29%) were older than 9 years; 22 children (24%) were included at a University Hospital, and 68 (76%) were included at a non-University center. Nine children (10%) completed only 1 day of the diary, and 10 children (11%) completed the diaries without all volume measurements but nevertheless were enough to be included in the data process.

We analyzed the incidence of MNE and NMNE diagnosis based on the CMT and/or voiding diary, subtyping according to the old and new ICCS definitions, and the diagnosis noted in the patient file. According to the new definition, NMNE is characterized by the presence of any LUTS, whereas the old one, only by urine loss (incontinence). Subtyping into MNE and NMNE revealed a difference depending on the ICCS standardization reference used and the diagnosis concluded by the clinician (Table 2). The physician’s diagnosis in the patient file generally agreed with the new ICCS definition. The diary at visit 2 revealed different prevalence percentages of NMNE and MNE.

**TABLE 2:** Enuresis subtyping based on the old and new ICCS definition and the patient note files.

	Old definition		New definition		Physician (patient file)	
	MNE	NMNE	MNE	NMNE	MNE	NMNE
CMT	45 (50%)	45 (50%)	15 (17%)	75 (83%)	18 (20%)	72 (80%)
Diary	66 (73%)	24 (27%)	16 (18%)	74 (82%)	31 (34%)	59 (65%)

*The prevalence of MNE is lower if the new definition is used.*

We divided the population into groups based on the documentation of LUTS symptoms at visits 1 and/or 2: MNE at both visits (MNE/MNE), NMNE at both visits (NMNE/NMNE), and different diagnoses at visits 1 and 2 (MNE/NMNE). As presented in Table 3, if the new ICCS definition is used for subtyping, the prevalence of MNE vs. NMNE is significantly lower (7 vs. 48%, respectively).

**TABLE 3:** Diagnosis groups based on the old and new ICCS definitions for NMNE. The prevalence of MNE is significantly lower when the new definition is applied.

	MNE/MNE	NMNE/NMNE	MNE/NMNE
Old definition ICCS	43 (48%)	22 (24%)	25 (28%)
New definition ICCS	6 (7%)	65 (72%)	19 (21%)

**FIGURE 1:**
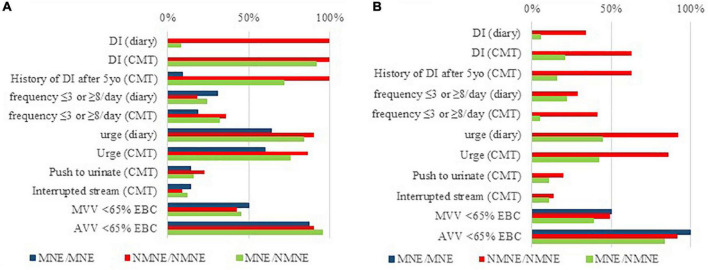
Prevalence of LUTS and small voided volumes when subtyping enuresis with regards to the old **(A)** and new **(B)** ICCS definition for NMNE, in the three studied clinical groups (MNE/MNE, NMNE/NMNE, and MNE/NMNE). Considering the new ICCS definition, it is urgency rather than actual daytime urine loss that leads to an NMNE diagnosis. Moreover, small voided volumes (MVV < 65% of EBC and AVV < 65% EBC) are equally present in MNE and NMNE patients, regardless of the ICCS definition used.

Figure 1 demonstrates the presence of the different LUTS, including maximum voided volume (MVV) and average voided volume (AVV) when smaller than the expected bladder capacity (EBC) for age {as defined by Hjälmås formula [EBC = (age + 1) × 30 ml]}. The presence of MVV and AVV smaller than 65% of the EBC [bladder capacity < 65% of EBC is considered small by the ICCS (2)] did not differ between MNE and NMNE regardless of the definition used. In addition, urgency rather than daytime incontinence (DI) was prevalent in the NMNE population when the new definition was used. Not only DI (CMT and diary) but also a history of DI after the age of 5 years old was statistically significantly different (*p* < 0.001) between the old and new ICCS definitions.

We observed considerable inconsistencies between the CMT and the diary for urgency (κ = 0.219), daytime incontinence (κ = 0.432), and especially for abnormal voiding frequency (≤3 or ≥8 voiding’s per day) between what the parents answered on the CMT and what they registered in the diary (κ = –0.057). We noted a strong correlation for the diagnosis made by the CMT and the diary for MNE (Spearman’s rank-order correlation [(*rs* = 0.612, *p* = 0.001) but not for NMNE (Spearman’s rank-order correlation not statistically significant (*rs* = 0.127, *p* = 0.248)].

Subsequently, we divided the children into groups based on the CMT and voiding diary diagnosis to perform further analyses: (a) MNE/MNE, (b) NMNE/NMNE, and (c) MNE/NMNE diagnosis group (the MNE/NMNE and NMNE/MNE groups are largely overlapping on characteristics except for the parameter urgency that is significantly higher in the CMT tool but is hardly ever registered in the diary (although requested). Since this is the only difference, the two groups were merged for further analysis). Daytime incontinence (at present and after 5 years of age) and urgency differed between the three groups as recorded in the CMT and in the diary (*p* < 0.001); abnormal voiding frequency was significantly different only as captured in the CMT (*p* = 0.003). When we investigated the groups individually, the registration of daytime incontinence differed significantly between the CMT and the voiding diary in the NMNE/NMNE group (*n* = 41 vs. *n* = 59, respectively, *p* < 0.001). The rest of the variables did not differ significantly between the three groups or separately.

**FIGURE 2:**
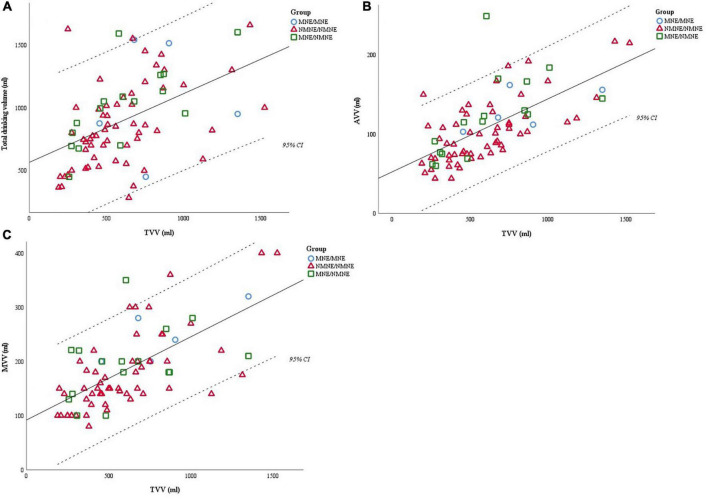
**(A)** Total drinking volume has a positive moderate correlation to the total voided volume (TVV); (*R* = 0.485, *p* < 0.001). **(B)** AVV (average voided volume) has a positive high correlation to the total voided volume (TVV); (*R* = 0.656, *p* < 0.001). **(C)** MVV has a positive high correlation to the total voided volume (TVV); (*R* = 0.656, *p* < 0.001). Total drinking volume is correlated with total voided volume (TVV), with the latter further correlating with the average voided volume (AVV) and maximum voided volume (MVV). Thus, we suggest that the registrations in the diary would be more reliable if the fluid intake was optimized before completing the voiding charts.

Furthermore, we investigated the voiding volumes as recorded in the diary. MVV was smaller than EBC in all groups (MNE/MNE *p* = 0.115; NMNE/NMNE *p* < 0.001, MNE/NMNE group *p* = 0.001). We observed a medium to a high positive correlation between the AVV, MVV, and Total Drinking Volume with Total Voided Volume (TVV) (Figures 2A–C). Specifically, we noted a moderate degree of correlation between the Total Drinking and the TVV [Pearson’s rank-order correlation statistically significant (*R* = 0.485, *p* < 0.001)] and a high degree of correlation between the MVV and TVV (Pearson’s rank-order correlation statistically significant [*R* = 0.656, *p* < 0.001)] and the AVV and TVV [Pearson’s rank-order correlation statistically significant (*R* = 0.656, *p* < 0.001)]. Children who drank more than 1,000 ml per day presented more frequently with daytime incontinence (*p* = 0.007) and had a higher TVV (*p* < 0.001). MVV and AVV were also higher but not statistically significant when corrected for age. Moreover, children with an abnormally low frequency (≤3 times per day) drank significantly less (*p* < 0.001) than the rest. Nine of ninety children had a voiding frequency of three voids/day at Visit 1 (of whom four had a fluid intake of < 600 ml) and 15/90 at Visit 2 (of whom five had a fluid intake of < 600 ml). Most of these children (7/9 and 13/15 for Visits 1 and 2, respectively) presented with daytime incontinence and/or urge, although incomplete registration in the diary cannot be excluded.

## Discussion

Recent ICCS guidelines state that subtyping enuresis in MNE and NMNE should not only be based on the presence or absence of daytime incontinence but also of the other LUTS, using both clinical history checklist (CMT) and registration in voiding diaries (1, 2). Our data show that most treatment-naïve patients have the NMNE subtype. The physician classifies only 20% of the patients primarily consulting for enuresis as MNE (1–3). However, rigorous analysis of the prospective registered LUTS symptoms (CMT + diary) shows that only 7% of the patients have any LUTS and do fulfill the strict criteria of MNE (Table 3). Clinicians give apparently subjective, expert opinion weight to the registered LUTS, in differentiating MNE from NMNE, not in line with the guidelines. It certainly reflects expert opinion that mild LUTS symptoms, other than daytime incontinence, does matter for the initial therapeutic approach.

Only a few studies have prospectively studied LUTS characteristics according to the different ICCS standardization papers. Subtyping is clinically vastly different in our population (Figure 1) with the presence of urgency rather than daytime urine loss (as registered in the CMT and diary) being predominant in NMNE as defined currently (1). Daytime incontinence and the history after the age of 5 years old were statistically significant for the two definitions and are very predictive for NMNE diagnosis at screening. The latter variable is a bad prognostic factor (18); thus, it should be registered at history taking and the CMT.

Our results showed a correlation between the diagnosis based on the diary and the one based on the CMT, but the two methods are not consistent for the type of enuresis (MNE or NMNE). CMT and the voiding diary do not have the same sensitivity nor specificity in identifying LUTS, therefore diagnosing NMNE. If we want to exclude any LUTS, then the two tools complement (19, 20).

In order to explain the inconsistencies between the two methods, we investigated the characteristics of our study population after dividing them into diagnosis groups (MNE/MNE, NMNE/NMNE, and NMNE/MNE-MNE/NMNE group). LUTS identification was inconsistent, leading to almost 30% of the children having a different diagnosis with the voiding diary compared to the CMT (Table 3). The significant higher incidence of daytime incontinence in the diary in the NMNE/NMNE group suggests that parents underestimate this symptom (21, 22).

This first Belgian national study describes the characteristics and incidence of MNE and MNE in naïve enuresis patients from expert as well as non-University centers. The use of CMT and voiding diaries, as recommended by the recent ICCS standardization paper, results in <10% of patients fulfilling the criteria of MNE, and even then, MVV and AVV are low for age. Norgaard et al. (23) used only diurnal incontinence for subtyping NMNE vs. MNE since this was associated with therapy resistance to the conventional monotherapy for MNE (desmopressin or alarm) (1, 2) in contrast with children with NMNE who might be referred to an expertise center. Documentation of LUTS other than daytime incontinence has decreased MNE incidence, although the evidence is lacking that this group could also not benefit from conventional therapy.

ICCS guidelines state that children with MNE can be managed with desmopressin and/or the enuresis alarm in a primary setting. This is largely based on studies prior to or following the first ICCS standardization paper (6). Evolving ICCS standardization papers have increased the sensitivity of identifying LUTS and therefore increased the diagnosis NMNE, but without evidence that this new NMNE-population would benefit more from a different first-line approach than the MNE.

Whether MVV and AVV are of significant importance in differentiating NMNE from MNE is questionable. MVV and AVV in all study groups were smaller than the bladder capacity expected for age, suggesting that estimation of voided volume in a diary may underestimate functional bladder volume (24). Notably, MVV and AVV correlate strongly with the total voided volume, and as such, with 24 h fluid intake (minus perspiration). This can be interpreted in two ways: children with small for age bladder volume will reduce fluid intake as natural prevention of frequency and urgency but, by that, undermining the sensitivity of the screening tool for LUTS. The other theory could indicate that most enuresis children have very low fluid intake during the daytime and should be enforced to increase fluid intake, which is conventionally advocated in most urotherapy guidelines. Our study does not allow any conclusion. Still, it suggests that a diary with or without a standardized/increased fluid intake will result in different CMT/diary data. Hence, probably the combination of both fluid intake methods is mandatory to get a complete interpretation for prospective studies on correlation with therapy response. It certainly would allow better stratification and identification of which children would benefit from a referral to an expertise center and a scientific point of view for a prospective study design. However, the increased complexity would make it less realistic and feasible in a primary care setting.

Our study has some limitations. Major criticism might be the relatively small cohort, but the findings are so conclusive that a larger cohort would not change the clinical significance. The added value of this study is that it is a multicenter one of treatment-naïve enuresis patients, primarily consulting for nighttime wetting in seven expert outpatient enuresis centers, all following common national standards for more than two decades and thereby warranting the quality of the data capturing, which would be difficult in a primary care setting. Moreover, the registration tool was explained and evaluated in person, not online. The majority of patients were recruited in non-university centers. A selection bias for refractory patients in the tertiary care center was eliminated since only treatment-naïve patients were included. There is some bias for the NMNE since we included only patients primarily consulting for nighttime symptoms and not for daytime symptoms (with enuresis). The higher incidence of bedwetting in boys than girls is in line with what is described in the literature. Still, the age distribution of these patients is somewhat higher than expected, now that information on the disease is so widely available online.

The studied CMT tool is limited in questions, therefore delivering minimal information on relevant comorbidities, known to coincide with therapy resistance: several more exhaustive questionnaires are available for evaluation of children’s incontinence (25–28), but to our defense, an all-inclusive questionnaire would be too long to serve as a screening tool. The registration of total voided volume did not include the nighttime output, as there is no country consensus policy regarding the registration of nocturnal diuresis (i.e., weighing the diapers in the morning) at the screening. Nevertheless, nocturnal diuresis volume is not crucial in differentiating MNE from NMNE, but only in identifying nocturnal polyuria and favoring desmopressin as first-line therapy. Lastly, MVV was considered the highest daytime volume. Previous studies have shown that MVV without the first morning voided volume correlates with age (29, 30) and more strongly with the EBC as defined by the Hjälmås formula (29), even though MVV with the first voiding volume included is higher than daytime MVV on the dry nights (31). MVV, as registered in voiding diaries, may be reliable in MNE (32) and children with LUTS (33) compared to volumes measured with other methods (uroflowmetry and cystoscopy).

## Conclusion

In conclusion, this study demonstrates that most patients with enuresis have some LUTS symptoms and should therefore be classified as NMNE. It confirms the inconsistencies between the CMT and the voiding diary in differentiating MNE from NMNE, where the data are complementary. Moreover, it gives further evidence that NMNE with all LUTS present and MNE without any LUTS are two archetypes on both ends of the spectrum and that many patients have only some of the symptoms, which, depending on fluid intake, can be masked or be apparent. Therefore we might conclude that any LUTS can only be excluded when diary registration and clinical history are performed during standardized fluid intake. On the other hand, it certainly questions whether LUTS other than daytime incontinence really matters in the first-line therapy. It seems too early to put the CMT tool in doubt (34). There is an urgent need to document if the patients with NMNE, formerly falling within the MNE group, are actually more refractory to first-line therapies (alarm/desmopressin).

## Data Availability Statement

The raw data supporting the conclusions of this article will be made available by the authors, without undue reservation.

## Ethics Statement

The study protocol was approved by the ethics committee of all participating hospitals (Belgian register number B403201630372 19 December 2017), and was carried out in accordance with the principles of the Declaration of Helsinki and the GCP/ICH/GDPR guidelines. Written informed consent to participate in this study was provided by the participants’ legal guardian/next of kin.

## Author Contributions

SK: collected the data, performed the statistical analyses, performed the measurements, and wrote the first draft of the manuscript. NR, PH, VD, PV, NS, JW, LD, and AB: conceived the study, collected the data, and revised the manuscript. All authors contributed to the article and approved the submitted version.

## Conflict of Interest

JW is a member of the advisory board, invited speaker, and PI of industry-sponsored studies of Astellas and Ferring. The remaining authors declare that the research was conducted in the absence of any commercial or financial relationships that could be construed as a potential conflict of interest.

## Publisher’s Note

All claims expressed in this article are solely those of the authors and do not necessarily represent those of their affiliated organizations, or those of the publisher, the editors and the reviewers. Any product that may be evaluated in this article, or claim that may be made by its manufacturer, is not guaranteed or endorsed by the publisher.

## Abbreviations

AVV: Average voided volume
CMT: Clinical management tool
EBC: Expected bladder capacity = (age + 1)*30 (ml) with a maximum of 360 ml
ICCS: International Children’s Continence Society
DI: Daytime incontinence
LUTS: Lower urinary tract symptoms
MNE: Monosymptomatic enuresis
MVV: Maximum voided volume (ml)
NE: Nocturnal enuresis
NMNE: Non-monosymptomatic enuresis
TVV, Total voided volume (ml/day): enuresis urine volume not included.

## References

[1] Neveus T Fonseca E Franco I Kawauchi A Kovacevic L Nieuwhof-Leppink A Management and treatment of nocturnal enuresis-an updated standardization document from the International Children’s Continence Society. *J Pediatr Urol.* (2020) 16:10–9. 10.1016/j.jpurol.2019.12.020 32278657

[2] Austin PF Bauer SB Bower W Chase J Franco I Hoebeke P The standardization of terminology of lower urinary tract function in children and adolescents: update report from the standardization committee of the International Children’s Continence Society. *Neurourol Urodyn.* (2016) 35:471–81. 10.1002/nau.22751 25772695

[3] Neveus T Eggert P Evans J Macedo A Rittig S Tekgül S Evaluation of and treatment for monosymptomatic enuresis: a standardization document from the International Children’s Continence Society. *J Urol.* (2010) 183:441–7. 10.1016/j.juro.2009.10.043 20006865

[4] Caldwell PH Codarini M Stewart F Hahn D Sureshkumar P. Alarm interventions for nocturnal enuresis in children. *Cochrane Database Syst Rev.* (2020) 5:CD002911. 32364251 10.1002/14651858.CD002911.pub3 PMC7197139

[5] Forsythe WI Butler RJ. Fifty years of enuretic alarms. *Arch Dis Child.* (1989) 64:879–85. 10.1136/adc.64.6.879 2673056 PMC1792561

[6] Norgaard JP Rittig S Djurhuus JC. Nocturnal enuresis: an approach to treatment based on pathogenesis. *J Pediatr.* (1989) 114(Suppl. 2):705–10. 10.1016/s0022-3476(89)80885-6 2926585

[7] Marzuillo P Marotta R Guarino S Fedele MC Palladino F Capalbo D ‘Frequently recurring’ nocturnal polyuria is predictive of response to desmopressin in monosymptomatic nocturnal enuresis in childhood. *J Pediatr. Urol.* (2019) 15:166.e1-e7. 10.1016/j.jpurol.2018.11.004 30528650

[8] Fielding D. The response of day and night wetting children and children who wet only at night to retention control training and the enuresis alarm. *Behav Res Ther.* (1980) 18:305–17. 10.1016/0005-7967(80)90089-3 7436978

[9] Butler RJ Gasson SL. Enuresis alarm treatment. *Scand J Urol Nephrol.* (2005) 39:349–57. 16257835 10.1080/00365590500220321

[10] Song P Huang C Wang Y Wang Q Zhu W Yue Y Comparison of desmopressin, alarm, desmopressin plus alarm, and desmopressin plus anticholinergic agents in the management of paediatric monosymptomatic nocturnal enuresis: a network meta-analysis. *BJU Int.* (2019) 123:388–400. 10.1111/bju.14539 30216627

[11] Austin PF Ferguson G Yan Y Campigotto MJ Royer ME Coplen DE. Combination therapy with desmopressin and an anticholinergic medication for nonresponders to desmopressin for monosymptomatic nocturnal enuresis: a randomized, double-blind, placebo-controlled trial. *Pediatrics.* (2008) 122:1027–32. 10.1542/peds.2007-3691 18977983

[12] Rittig N Hagstroem S Mahler B Kamperis K Siggaard C Mikkelsen MM Outcome of a standardized approach to childhood urinary symptoms-long-term follow-up of 720 patients. *Neurourol Urodyn.* (2014) 33:475–81. 10.1002/nau.22447 23765698

[13] Franco I von Gontard A De Gennaro M International Childrens’s Continence Society. Evaluation and treatment of nonmonosymptomatic nocturnal enuresis: a standardization document from the International Children’s Continence Society. *J Pediatr Urol.* (2013) 9:234–43. 10.1016/j.jpurol.2012.10.026 23260268

[14] Austin PF Bauer SB Bower W Chase J Franco I Hoebeke P The standardization of terminology of lower urinary tract function in children and adolescents: update report from the Standardization Committee of the International Children’s Continence Society. *J Urol.* (2014) 191:1863–65.e13. 24508614 10.1016/j.juro.2014.01.110

[15] Vande Walle J Rittig S Bauer S Eggert P Marschall-Kehrel D Tekgul S. Practical consensus guidelines for the management of enuresis. *Eur J Pediatr.* (2013) 171:971–83. 10.1007/s00431-012-1687-7 22362256 PMC3357467

[16] Flynn JT Kaelber DC Baker-Smith CM Blowey D Carroll AE Daniels SR Clinical practice guideline for screening and management of high blood pressure in children and adolescents. *Pediatrics.* (2017) 140:e20171904. 28827377 10.1542/peds.2017-1904

[17] Nevéus T von Gontard A Hoebeke P Hjälmås K Bauer S Bower W The standardization of terminology of lower urinary tract function in children and adolescents: report from the standardisation committee of the international children’s continence society. *J Urol.* (2006) 176:314–24. 10.1016/S0022-5347(06)00305-3 16753432

[18] Yeung CK Sreedhar B Sihoe JD Sit FK Lau J. Differences in characteristics of nocturnal enuresis between children and adolescents: a critical appraisal from a large epidemiological study. *BJU Int.* (2006) 97:1069–73. 10.1111/j.1464-410X.2006.06074.x 16643494

[19] Kwak KW Park KH. Clinical inconsistency of lower urinary tract symptoms between questionnaire and bladder diary in children with nocturnal enuresis. *J Urol.* (2008) 180:1085–9; discussion 9–90. 10.1016/j.juro.2008.05.053 18639291

[20] Sajith S Patnaik SK Kanitkar M. Comparison of a voiding diary to clinical management tool for diagnosis of voiding disorders in children. *Pediatr Nephrol*. (2016) 31:1773. 34183463

[21] Schlomer B Rodriguez E Weiss D Copp H. Parental beliefs about nocturnal enuresis causes, treatments, and the need to seek professional medical care. *J Pediatr Urol.* (2013) 9(Pt B):1043–8. 10.1016/j.jpurol.2013.02.013 23608323 PMC4648250

[22] Sureshkumar P Caldwell PH Craig JC. Diagnosing daytime bladder symptoms in children with nocturnal enuresis: a comparison of brief parental questionnaire with in-depth, physician-elicited, assessment. *J Paediatr Child Health.* (2010) 46:636–41. 10.1111/j.1440-1754.2010.01821.x 20796179

[23] Nørgaard JP van Gool JD Hjälmås K Djurhuus JC Hellström AL. Standardization and definitions in lower urinary tract dysfunction in children. International children’s continence society. *Br J Urol.* (1998) 81(Suppl. 3):1–16. 10.1046/j.1464-410x.1998.00025.x 9634012

[24] Van Hoeck K Bael A Lax H Hirche H Van Dessel E Van Renthergem D Urine output rate and maximum volume voided in school-age children with and without nocturnal enuresis. *J Pediatr.* (2007) 151:575–80. 10.1016/j.jpeds.2007.05.023 18035133

[25] Chase J Bower W Gibb S Schaeffer A von Gontard A. Diagnostic scores, questionnaires, quality of life, and outcome measures in pediatric continence: a review of available tools from the International Children’s Continence Society. *J Pediatr Urol.* (2018) 14:98–107. 10.1016/j.jpurol.2017.12.003 29429829

[26] Drzewiecki BA Thomas JC Pope JCT Adams MC Brock JW III Tanaka ST. Use of validated bladder/bowel dysfunction questionnaire in the clinical pediatric urology setting. *J Urol.* (2012) 188(Suppl. 4):1578–83. 10.1016/j.juro.2012.02.036 22910262

[27] Anwar T Cooper CS Lockwood G Ferguson KJ Barlow PB Storm DW. Assessment and validation of a screening questionnaire for the diagnosis of pediatric bladder and bowel dysfunction. *J Pediatr Urol.* (2019) 15:528.e1-e8. 10.1016/j.jpurol.2019.07.016 31445857

[28] Altan M Citamak B Bozaci AC Mammadov E Dogan HS Tekgul S. Is there any difference between questionnaires on pediatric lower urinary tract dysfunction? *Urology.* (2017) 103:204–8. 10.1016/j.urology.2016.12.055 28082122

[29] Rittig S Kamperis K Siggaard C Hagstroem S Djurhuus JC. Age related nocturnal urine volume and maximum voided volume in healthy children: reappraisal of International Children’s Continence Society definitions. *J Urol.* (2010) 183:1561–7. 10.1016/j.juro.2009.12.046 20176383

[30] Kim SO Kim KD Kim YS Kim JM Moon du G Park S Evaluation of maximum voided volume in Korean children by use of a 48-h frequency volume chart. *BJU Int.* (2012) 110:597–600. 10.1111/j.1464-410X.2011.10799.x 22145861

[31] Cho WY Kim SC Kim SO Park S Lee SD Chung JM Can recording only the day-time voided volumes predict bladder capacity? *Investig Clin Urol.* (2018) 59:194–9. 10.4111/icu.2018.59.3.194 29744477 PMC5934282

[32] Maternik M Chudzik I Krzeminska K Żurowska A. Evaluation of bladder capacity in children with lower urinary tract symptoms: comparison of 48-hour frequency/volume charts and uroflowmetry measurements. *J Pediatr Urol.* (2016) 12:214.e1-e5. 10.1016/j.jpurol.2016.04.004 27329866

[33] Uluocak N Oktar T Ander H Ziylan O Acar O Rodoplu H Which method is the most reliable in determination of bladder capacity in children with idiopathic overactive bladder? A comparison of maximum voided volume, uroflowmetry and maximum cystometric capacity. *J Pediatr Urol.* (2009) 5:480–4. 10.1016/j.jpurol.2009.03.002 19342301

[34] Fuentes M Magalhães J Barroso U Jr Diagnosis and management of bladder dysfunction in neurologically normal children. *Front Pediatr.* (2019) 7:298. 10.3389/fped.2019.00298 31404146 PMC6673647

